# ‘It's all there in black and white’ – or is it? Consumer perspectives on the proposed Australian Medicine Information Box over‐the‐counter label format

**DOI:** 10.1111/hex.12389

**Published:** 2015-07-31

**Authors:** Vivien Tong, David K. Raynor, Parisa Aslani

**Affiliations:** ^1^ Faculty of PharmacyThe University of SydneySydneyNSWAustralia; ^2^ School of HealthcareUniversity of LeedsLeedsUK

**Keywords:** consumers, drug labelling, non‐prescription medicines, over‐the‐counter, self‐management

## Abstract

**Background:**

Over‐the‐counter (OTC) labels help support safe medication use by consumers. In 2012, the Australian Therapeutic Goods Administration (TGA) released a consultation paper proposing OTC label standardization to improve label quality via implementation of the Medicine Information Box (MIB) label. However, consumer opinions of the MIB and standardization of OTC labelling remain unexplored.

**Objective:**

To explore consumer perspectives of OTC label standardization and the proposed MIB.

**Design:**

Mock MIB labels were developed by the research team, guided by the TGA consultation paper, and used as interview stimulus material.

**Participants and setting:**

Semi‐structured interviews were conducted with 38 Australian and 39 UK adult participants. Participant perspectives on OTC label standardization, opinions on the MIB and perceived improvements were explored. All interviews were audio‐recorded with permission, transcribed verbatim, and the content thematically analysed.

**Results:**

Participants expressed a range of opinions towards OTC label standardization, from welcoming standardization to concern that important details may be overlooked. The MIB was generally positively received due to its perceived good information design and ease of navigation. Participants requested reordering of information‐specifically, for the active ingredient to be moved to a less prominent position. Suggested improvements centred on content and design changes, for example colour, pictograms, bolding.

**Conclusions:**

Participants felt positively towards OTC label standardization and saw the MIB as a feasible standardized format to implement for OTC labels. Although they appreciated its good information design, they felt further improvements to its content and design are required to enhance its quality and usability.

## Introduction

Over‐the‐counter (OTC) medicines provide consumers with the important opportunity to actively engage in self‐management. From a medication safety perspective, information provided with OTC medicines must be relevant, user‐friendly and also adequately meet consumer information needs. Pharmacists,^1^, ^2^, ^3^ patient information leaflets^1^, ^4^ and product labels^1^, ^5^, ^6^ are noted OTC medicine information sources for consumers. With respect to health‐care professionals, pharmacists are regarded by consumers as a primary OTC medicine information source.^3^ However, spoken information exchanged between pharmacy personnel and consumers can vary,^7^, ^8^ and sometimes adequate or appropriate information may not be exchanged. Consumers may not always discuss their OTC medication use with their doctor^9^ or remember all the advice provided by pharmacy staff.^10^ In the European Union, all medicines (including OTC medicines) must have a patient information leaflet included as a package insert.^11^ In Australia, however, differences in OTC medicine scheduling^12^ lead to variations in leaflet availability. With respect to OTC medicines, Consumer Medicine Information (CMI) leaflets must be available only for all Schedule 3 Pharmacist‐only medicines (medicines provided only in pharmacies with pharmacist involvement^12^)^13^; hence, leaflets are not mandatory for Schedule 2 Pharmacy medicines (medicines available in pharmacies, where an opportunity to obtain advice from pharmacy staff is available where necessary^12^). Leaflets therefore serve as a useful additional OTC medicine information source.

Product labels are a source of information accompanying all OTC medicines. They have a high consumer readership^14^, ^15^ and are influential in aiding consumers in treatment decision making.^5^ OTC labels are highly accessible, consistent and medicine‐specific, and potentially accompany medicines throughout their use by consumers. Furthermore, as leaflets are not available with some OTC medicines in Australia, the label becomes increasingly important.

High OTC label quality and usability are central to maximizing medication safety. However, labels may not always adhere to guidelines incorporating design recommendations,^16^, ^17^ and variable consumer understanding of OTC labels has been noted.^18^ Where OTC labels cannot be satisfactorily understood, this can increase the likelihood of patient harm. Different regulatory environments have explored various strategies to improve OTC labelling quality for consumers, such as the recommendation for ‘user testing’ in OTC label development and testing as part of the Labelling Code of Practice in Australia,^19^ and the standardization of OTC labels in the United States (U.S.) with the Drug Facts label format.^20^ Two large‐scale studies, that explored consumer preferences and testing of the Drug Facts label format versus older OTC labels, supported the legislation of OTC label standardization in the U.S.^20^ The Drug Facts label format appeared to improve the time taken for consumers to locate information^20^, ^21^, ^22^ in comparison with older OTC labels.

At present, regulatory environments such as Australia and the European Union have not implemented OTC label standardization. In Australia, a number of legislative documents outline OTC labelling requirements,^23^ where specifically, the Therapeutic Goods Order No. 69 provides detailed content‐focussed requirements.^24^ However, a consultation paper released in 2012 by the Australian Therapeutic Goods Administration proposed the Medicine Information Box (MIB) OTC label format, in a bid to introduce regulatory change to support standardization of OTC labels in Australia.^25^ Its introduction was intended to increase the ease of locating critical medicine information and support safe and appropriate medication use.^25^

At present, there is limited evidence available on consumer opinions about the MIB or its usability and therefore little evidence to support standardization using the MIB. In addition, there is a lack of international comparative data in relation to consumer opinions on proposed OTC label standardization within different regulatory environments that have yet to adopt this labelling strategy. Therefore, the aim of this study was to explore consumer perspectives on the MIB and OTC label standardization in Australia and the United Kingdom (UK).

## Methods

These interviews formed part of a broader international research project, which examined consumer use, understanding and perspectives on OTC medicine information in Australia and the UK. The methods for this study involved three stages: mock MIB label development, semi‐structured interviews and data analysis.

### Development of mock MIB labels

**Figure 1:**
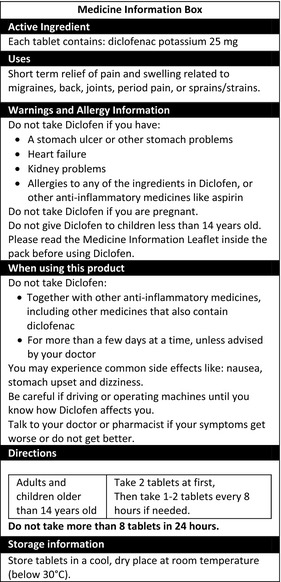
MIB developed by the research team for diclofenac (presented on a panel with dimensions 70 mm × 154 mm).

**Figure 2:**
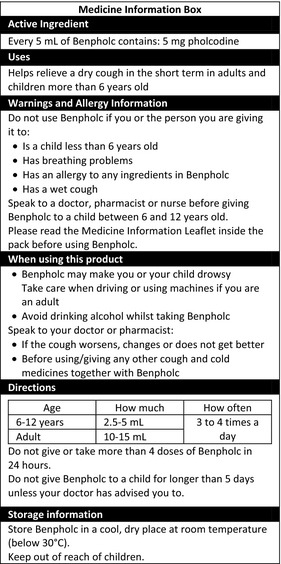
MIB developed by the research team for pholcodine (presented on a panel with dimensions 65 mm × 150 mm).

Two mock MIB labels for OTC medicines diclofenac (Fig. 1) (available as a Schedule 3 Pharmacist‐only medicine in Australia) and pholcodine (Fig. 2) (available as a Schedule 2 Pharmacy medicine in Australia) were developed, with reference to the MIB published in the Australian TGA consultation paper.^25^ These medicines were chosen as exemplars of products available in two Australian OTC medicine schedules. Both are available as Pharmacy Medicines in the UK (available from a pharmacy without a prescription, but under pharmacist supervision).^26^ The MIB headings, order of headings, and black and white format included in the Australian TGA consultation paper^25^ were used as the framework.

Relevant clinical content for each exemplar medicine was included within the framework, and based on OTC medicine information available with one existing Australian and one UK proprietary product with the same active ingredient [diclofenac: Voltaren^®^ Rapid 25 (Novartis Consumer Health Australasia Pty Ltd, Mulgrave, VIC, Australia), Voltarol Pain‐eze^®^ Extra Strength 25 mg tablets (Novartis Consumer Health, Horsham, UK); pholcodine: Benadryl^®^ Dry, Tickly Cough (Johnson & Johnson Pacific Pty Ltd, Ultimo, NSW, Australia), Benylin^®^ Children's Dry Coughs (BCM Nottingham, Nottingham, UK)]. To help facilitate MIB content completeness, key content points were pooled and agreed upon for inclusion by all research team members. Reference texts such as the Australian Pharmaceutical Formulary and Handbook (22nd edition)^27^ and Australian Medicines Handbook (2012)^28^ were also consulted. Plain English and good information design principles^29^ were utilized during development (whilst keeping the overall proposed MIB intact). All research team members reviewed and contributed to the development of the labels. Once finalized, they were affixed to blank cardboard boxes to simulate the inclusion of the MIB on the back panel of the product packaging if implemented.

### Semi‐structured interviews

Semi‐structured interviews were chosen as the qualitative method to explore consumer perspectives on the MIB, as it afforded the interviewer a degree of flexibility to diverge from the protocol where necessary to gain further insight into the perspectives raised^30^; this was of particular use as there has been limited research completed in the area to date. Interviews were conducted between April 2013 and April 2014 with 40 Australian and 40 UK participants in Sydney, Australia, and Leeds, UK, respectively. However, due to time constraints, only 38 and 39 of the 40 Australian and 40 UK semi‐structured interviews, respectively, addressed the current study objectives.

#### Recruitment

Australian participants were recruited through the distribution of recruitment flyers, online advertisements and by a market research company. UK participants were recruited through the use of a consumer database maintained by Luto Research, a company which offers health information user testing services in Leeds, UK. Participants were eligible for inclusion if they were:

1. aged 18 years and above;
2. conversant in English (did not require a translator to participate);
3. had purchased and used an OTC medicine (for themselves or had given it to someone under their care) in the 6 months prior to study participation (but not the exemplar study medicine within this period; or a similar medicine in the 3 months prior to study participation); and
4. had not participated in a user testing study within the 6 months prior to study participation (criterion specific to the UK study arm).

Participants were excluded if they:

1. were a retired or practising health‐care professional;
2. currently employed in an occupation which primarily dealt with medicine information; or
3. had significant visual or cognitive impairment that would affect their participation in the study.

**Table 1:** Summary of participant demographics

Demographic	Australia (*n* = 38)	UK (*n* = 39)	Total
Gender			
Male	19	19	38
Female	19	20	39
Age			
18–29	10	8	18
30–49	14	14	28
50–69	12	10	22
70+	2	7	9
Highest level of education			
School Certificate/GCSE^a^ (Year 10) or below	1	10	11
Higher School Certificate/A Level^b^ (Year 12) or College qualification	26	21	47
Bachelor degree or above	11	8	19
Main language spoken at home			
English	35	39	74
Other	3	0	3
Country of birth			
Australia	32	1	33
UK	1	36	37
Other	5	2	7

a GCSE – UK General Certificate of Secondary Education.

b A level – UK General Certificate of Education Advanced Level.

Table 1 summarises the participant demo‐graphics.

#### Study process

All Australian and UK semi‐structured interviews were conducted by one female researcher (VT) on site at The University of Sydney, Australia, or Luto Research, UK. The entire face‐to‐face session lasting about 1 h was broadly structured as follows:

1. ‘User testing’ of an OTC label and leaflet:

‘User testing’^31^ examined written medicine information performance via the assessment of how well participants were able to find and understand key points of information. Medicine information accompanying Australian and UK diclofenac and pholcodine proprietary products was ‘user tested’ by participants in both countries.

Semi‐structured interview:

By conducting the semi‐structured interview immediately after the ‘user testing’, participants were exposed to two different OTC label formats for products with the same active ingredient for comparative purposes: an existing OTC label and the corresponding MIB. Following user testing and feedback on the existing label, participants were given a printed copy of the corresponding MIB and asked for their:

- opinions on the MIB;
- views on standardization of OTC labels;
- preferences between the existing label and corresponding MIB examined; and
- perceived improvements required to optimize the MIB.

Participants were reimbursed for their time associated with study participation.

This paper focuses on consumer perspectives on the MIB and OTC label standardization in Australia and the UK.

### Data analysis

Interviews were audio‐recorded with permission and transcribed verbatim. Transcripts were checked against the audio recording by the interviewer (VT) to ensure transcription accuracy and data familiarization. Checked transcripts were then thematically content analysed.^32^ Data analysis was primarily conducted by one researcher (VT). Relevant subthemes derived from the data were consolidated and grouped under identified relevant broad themes within each data set (Australia and UK). Independent thematic content analysis of a portion of both Australian and UK transcripts by a second researcher (PA) was completed to assess validity of data analysis and reliability of identified themes and subthemes. Themes and subthemes for each data set were displayed concurrently using a matrix display,^33^ to allow for ease of comparison and contrast between the Australian and UK findings, which was reviewed and discussed by the research team members. Thematic or data saturation^34^ was achieved within each Australian and UK interview data set.

Ethics approval for the study was granted by the University of Sydney Human Research Ethics Committee, Sydney, Australia, and the School of Healthcare Research Ethics Committee, University of Leeds, UK.

## Results

A total of 37 (18 Australian, 19 UK) and 40 (20 Australian, 20 UK) participants provided feedback on the diclofenac and pholcodine mock MIBs, respectively. Overall, both Australian and UK interview findings were similar and have been combined. However, any significant differences between the two interview cohorts or findings unique to a specific cohort will be emphasized where appropriate.

Participants were broadly in favour of the MIB as a way of standardizing OTC labels. Participants voiced appreciation for MIB aspects which converged around core good information design concepts. However, visual attractiveness of the MIB was seen to be a key area requiring improvement. Many suggestions to improve the quality and/or usability were proposed, which have been presented below.

### Broad consumer opinions regarding OTC label standardization

Standardization was seen by many participants as a positive approach overall, where it could enable familiarization when locating information on the label.

Yes, I think it would be better if there was some sort of standardized format for, for leaflet and information on packets. Um … ‘cause obviously, the longer the, the standardized formats are around, people would get much more used to reading it and finding what they need to know quickly. (UTP71‐UK)

Particularly, in the UK cohort, standardized OTC labels had the potential to accelerate information retrieval, reported as useful for ease of initial label navigation. Standardization was also perceived to be useful to locate information across different products. Some also compared standardization to nutrition labelling (where in Australia, for example, a standardized format has been adopted^35^).

Conversely, an Australian participant raised that standardization may encourage overlooking of medicine‐specific information between products (such as dosage regimens) due to the uniform layout and, thus, may contribute to incorrect use.

My first reaction is yes, but I hesitate because if every medicine looks the same, … it might be that we get complacent; for example, it might be a different dosage between two different medicines … if everything looked the same, we might be tempted to, um, be lazy about it and not read the instructions properly. (UTP26‐AUS)

With a detailed label such as the MIB, one UK participant also commented that it may ‘tempt me not to read the [patient information] leaflet [found inside the box]’ (UTP41‐UK).

Other participants were ambivalent towards standardized labels. Standardization was seen to make minimal difference for irregular OTC medicine users; for instance, an Australian participant explained that information would be read regardless of the label format/design because they were not using a product regularly. An UK participant commented that MIB re‐evaluation post‐implementation would be necessary to review its effectiveness in meeting consumer needs.

I've got everything I need, I can see this, this is great……it has its benefits because it's straight to the point, and I can pick out key information. Whether that would need to change, it depends on … each individual case and the person's needs. But for me personally… It does what I would like it to do. If I received all my medication like this for a period of six months, then I think I'd be in a better position to suggest improvements.’ (UTP58‐UK)

### Consumer impressions of the MIB

Overall, mixed views regarding the MIB were demonstrated within both participant cohorts. Australian participants on the whole preferred the MIB; however, some still preferred the existing labels. This was similar for the UK cohort; however, more UK compared to Australian participants preferred the existing label that they had user tested over the corresponding MIB. A few participants saw little difference between existing and MIB labels.

Medicine Information Box content appeared to encompass core OTC medicine information needs for participants, for example dosage, uses, contraindications.

It has all of the basic information that you need straight away. Storing it, you know, taking it, when not to take it. That kind of thing. (UTP11‐AUS)

Some participants liked the detailed information contained in the MIB, where extra information outweighed visual appeal in this instance. In particular, an Australian participant (mother of a young child with asthma and allergies) perceived the pholcodine MIB to contain more information that suited her needs as a parent in comparison with the Benadryl^®^ Dry, Tickly Cough label (i.e. warnings information).

Similarly, a few participants preferred the existing Benadryl^®^ Dry, Tickly Cough label for colour/appearance; however, the corresponding MIB was preferred for content. Conversely, others thought that the MIB content was too much. Overall, UK participants felt it represented potential content duplication with the package insert.

Some participants felt packaging size may potentially impact MIB implementation on all current OTC labelling. In particular, the packaging size difference between the smaller existing diclofenac proprietary product and diclofenac MIB was commented on. Many participant responses reflected an acknowledgement, whether directly or indirectly, of an interrelationship between design, content, and/or usability of the MIB.

I don't know, because … there's a limit to the amount of information that can be contained on here, without making it, without destroying its attractiveness and clarity, isn't there? (UTP76‐UK)

### Positive aspects of the MIB

Participants commented on many positive aspects of the MIB (Table 2). Overall, many perceived the MIB to be easier, simple and more accessible than the existing labels they had user tested.

**Table 2:** Summary table outlining positive and negative aspects of the MIB

Positive aspects of the MIB	Negative aspects of the MIB
Easier, simpler and improved accessibility	Unattractive, black and white format
Clearer and improved format	Negative emotional response instilled by MIB (‘fear’)
Ordered presentation of information	Difficulty in reading (attributed to black and white format)
Use of bullet points and clear headings	Information design expertise more clearly demonstrated in existing label
Good sectioning of information	
Tabulated directions for use	
Larger font size

Each subsection highlighted as bold as, as brass and ah, everything is sort of covered, I, I would expect to be covered on the box really. (UTP73‐UK)

The black font and white background was reported to be easier to read by some. Bullet points and headings were positively received and seen to contribute to the user‐friendly nature of the MIB. Good sectioning of information provided ‘clear demarcation between them’ (UTP46‐UK). Tabulated directions for use were preferred by some participants.

### Negative aspects of the MIB

Participants identified some negative aspects of the MIB (Table 2). Both Australian and UK participants reported that the black and white format was unattractive and ‘not as… easy on the eye’ (UTP64‐UK). The absence of colour contributed to its perceived unattractiveness. A few participants also felt the black and white format was harder to read. It was seen to instil ‘fear’ by a few participants, where, for example, one UK participant stated that it seemed like ‘you're drinking anthrax, highly toxic, dangerous product’ (UTP74‐UK).

An UK participant suggested that information design expertise was more evident in the existing Benadryl^®^ Dry, Tickly Cough label, as opposed to the corresponding MIB.

Well, it needs to be presented in similar form to this [Benadryl^®^ label]. This has been very carefully written and designed to cover all the important points without the need for a pamphlet, hasn't it? And, uh, also someone with a knowledge of what sort of presentation of information the man or woman in the streets are likely to take in quickly and precisely. This [MIB] is harder work, for some reason, to read than this [Benadryl^®^ label]. (UTP76‐UK)

### Desired measures to improve the MIB

Participants proposed a number of desired improvements that would help contribute to higher perceived MIB quality and usability (Table 3).

**Table 3:** Desired measures to improve the MIB

Desired improvement	Specific suggestions/examples^a^
Reordering of information and/or headings	Include directions for use in position of higher prominence
	Move active ingredient to a less prominent position
Colour	Coloured fonts, background(s)
	Colour coding of different sections (UK)
	Convey warnings using red
Bolding for emphasis	Use bolding to emphasize warnings information
	Bold ‘not’ in ‘Do not…’
	Bold key precautions/contraindications terms (Australia)
	Bold subheadings (Australia)
Picture or pictogram use	‘Tick cross’ pictogram system
Content	Content addition: contact details (Australia), inactive ingredient information, expiry date (UK)
	Omit active ingredient from MIB
	Content reduction (UK): ‘when using this product’ information, some actions required to be taken (statements referring to speak to a health‐care professional or read the enclosed leaflet), ‘Storage’ heading
Rewording of headings	Headings should be direct statements corresponding to action to be taken (Australia)
	‘Warnings and allergy information’ to be replaced with: ‘Precautions’ (perceived to be more friendly than ‘Warning’) (UK)
	‘Directions’ to be replaced with: ‘Dosage’ (UK); ‘How to take this medicine’ (Australia)
	‘When using this product’ to be replaced with: ‘Attention’ or ‘Warning’ (UK); ‘Do not take/use if’ (Australia)
Other formatting changes	Split MIB across multiple panels
Use of a thin line to subdivide subsections under one heading (Australia)
Distinct, separate sections similar to the Benadryl^®^ Dry, Tickly Cough label (UK)

a Suggestions were provided by both Australian and UK cohorts, unless specified otherwise.

#### Reordering of information and/or headings

Participants’ desire for positioning and ordering of information and/or headings appeared to reflect considerations such as: recognition of logical, safety‐inclined information ordering or; preference for information to occupy a position corresponding to its perceived relative importance. For instance, ‘Directions for use’ were suggested by some participants to be included in a more prominent position (i.e. higher up) due to its perceived importance, as reflected in the positioning of ‘Uses’ information in the MIB.

I mean, I would put the main information, like the uses‐ and how to take it, first…… because that's the first two things someone would want to know. And then, the cautions because, obviously, then you want to know, right, what am I cautious about? Am I taking anything else? Or do I not take it with this and that? Um, so, I'd just change that around. (UTP39‐AUS)

Notably, active ingredient information was viewed by many Australian and UK participants as less important in comparison with information such as directions for use. It did not constitute a salient information need other than in specific situations, such as intentional active ingredient avoidance (e.g. drug interactions), and thus, was not commonly actively sought.

The current position of prominence occupied by active ingredient information did not reflect its relative perceived weighted importance. Consequently, participants requested it to be moved to a less prominent position, that is lower down in the MIB.

#### Colour

Colour integration into the MIB was a widespread desire for Australian and UK participants, viewed as a way to improve its visual attractiveness.

This is a help, not a hindrance. So … if it's a help, then make it look more helpful… then colour it. (UTP03‐AUS)

Various specific suggestions as to how colour could be incorporated were put forward (Table 3). A few participants appeared to be influenced by colour schemes adopted by the existing user tested label and hence suggested a similar colour scheme for the MIB (such as blue and white used in the Benylin^®^ Children's Dry Coughs label). A popular suggestion across both cohorts was for warnings to be conveyed using the colour red. Some Australian participants also commented that the colour(s) should be appropriate for the colour blind.

#### Bolding for emphasis

Australian and UK participants called for bolding to help emphasize warnings information (Table 3); for example, to ‘highlight… the more dangerous items on there in… bold print’ (UTP67‐UK). Consequently, bolding of ‘not’ in ‘Do not…’ was desired. A few Australian participants desired key precautions/contraindications terms to be bolded, such as ‘pregnant’.

#### Pictures or pictogram use

Pictures or pictogram use in the MIB were potential improvements raised by a few Australian and UK participants.

And I've done a lot of stuff with people with disabilities. And pictures always speak a thousand words more than words do. People get a bit confused by words or a bit intimidated by words, actually. (UTP09‐AUS)

The ‘tick cross’ pictogram system (utilized on the Benadryl^®^ Dry, Tickly Cough label, where ticks and crosses replace conventional bullet points to reinforce indications and contraindications/precautions, respectively) was favoured for use by a few participants.

#### Content

A small number of participants requested additional content (Table 3) such as: a toll‐free information line/emergency contact details (Australia) and complete inactive ingredient information (Australia) [or to a similar extent as per the existing label read (UK)].

In contrast, UK participants requested deletion of certain content (Table 3). An UK participant proposed deletion of the ‘Storage’ heading, with storage information to be included at the bottom without a separate heading, as it was not seen as important enough to warrant a separate heading. A few participants (Australia and UK) suggested omission of the active ingredient from the MIB and potentially included elsewhere, such as on the principal display panel or in the leaflet.

#### Rewording of headings

A few participants were not completely satisfied with the wording of the MIB headings (Table 3). For instance, one Australian participant explained that as they did not suffer from allergies, other warnings included under the ‘Warnings and allergy information’ heading were overlooked.

So, ‘Warnings and allergy information’… I've sort of overlooked that as well, because um, I know that I don't have any allergies straight away. However, you know… sometimes I have some stomach problems. So, I've already missed that [warning] before, because I think “I'm not really allergic to anything”… So I would say… the heading may need to be changed a little bit. (UTP30‐AUS)

## Discussion

A wide spectrum of participant opinions on the MIB was evident, in particular regarding perceived improvements required. However, overall, most participants positively received OTC label standardization as a potential labelling strategy. Thus, standardization has the potential to meet consumer OTC medicine information needs and improve OTC label quality, as demonstrated by previous testing of the U.S. Drug Facts label format^20^ and in particular for younger consumers.^21^, ^22^ Despite promising evidence for standardization, conveying information using the Drug Facts label format does not completely safeguard against consumer misunderstanding of critical OTC medicine information.^36^ Consequently, it is imperative to involve consumers in refining and testing the MIB (whose format was based on the Drug Facts label^25^) in preparation for implementation, as seen by the breadth of suggested improvements.

Participant perspectives regarding OTC label standardization in many respects corresponded with the rationale for introduction of the MIB, as stipulated in the Australian TGA consultation paper.^25^ Participants appreciated aspects of the MIB which mirrored good information design principles, such as the use of bullet points and appropriate, clear headings,^29^ and effective use of tables (by tabulating directions for use). Similar to the present study, it has been demonstrated that consumer preference exists for labels with increased white space and font size.^37^, ^38^ This alignment of good information design principles and participant perceptions of the positive aspects of the MIB is promising and reinforces the value of integrating good information design when developing written medicine information. Despite a higher weighting placed on detailed content over visual appeal by some, visual appeal of the MIB was seen as lacking by many. As such, improving visual attractiveness is one of many pragmatic identified improvements that can boost consumer receptiveness of the MIB if addressed. This could lead to increased likelihood of consumers reading OTC labels and thereby enabling the retrieval of necessary medicine information to support their safe use. Innovative characteristics incorporated into existing labelling, such as the ‘tick cross’ pictogram system utilized on the Benadryl^®^ Dry, Tickly Cough label, should be considered when developing a standardized label format. Furthermore, simple strategies such as the introduction of at least one colour into the MIB may decrease perceived unattractiveness and better cater for consumers’ needs, whilst still ensuring good information design aspects remain intact to support medication safety. The integration of colour versus a black and white format should also be explored further, particularly in light of mixed perceived quality of readability of black font on a white background.

The ‘Directions for use’ section was highly valued by participants, as shown previously.^1^ Broad information ordering preferences seen in the present study, for example, the inclusion of information such as indications, directions and warnings before active ingredient on the label, were similar to previous overall study findings.^39^ The FDA study conducted to ascertain consumer label preferences also noted that the inclusion of directions higher up in the label was one of the more commonly cited reasons for preferring a label format.^40^ Following on from this apparent trend in information ordering preferences, prior to MIB finalization, it may be worthwhile to further explore alternative ordering of MIB headings in an attempt to better cater for preferences of the consumer population at large. However, there is the potential for a disconnect between consumer preference and performance‐based usability^41^ which should be acknowledged and considered in the refinement process.

The conscious inclusion of active ingredient information at the top of the MIB acknowledges its importance in improving medication safety for consumers from a professional and scientific development perspective. Similar to the present study findings, King *et al*.^42^ found that consumers did not commonly focus on the active ingredient unless there was an awareness of the need to avoid concomitant use with other medicine(s) being used. Interestingly, labels that included the active ingredient lower down have been shown to be more preferred by consumers than labels with active ingredient presented first,^40^ and yet the implemented Drug Facts label still includes the active ingredient foremost.^20^ This disparity in perceived importance from the consumer perspective may reduce the intended significant impact of this pre‐designated information order, such as that seen in the proposed MIB. The shifting of the active ingredient to the bottom of the label was also discussed in the expert comments made in response to the TGA consultation paper.^43^ Accordingly, consideration should be given to relocating active ingredient information to a less prominent position in the MIB. However, with indications of a mismatch between participants’ desired information ordering and the current MIB, when moving forward, it is critical to ascertain whether catering for consumer needs has implications on label performance and medication safety, such as in the case of the proposed omission of the active ingredient from the MIB as one example.

Alongside this, further public health efforts to better inform consumers of key aspects integral to medication safety to better support the purpose of the implementation of the MIB should be considered; for example, active ingredient awareness. One example of such an initiative is the information provided by NPS MedicineWise, a not‐for‐profit organization that provides numerous resources for consumers regarding health and medicines, which encourages and explains to consumers the importance of active ingredient identification.^44^

With respect to the proposed MIB, an important caveat is that evidence to demonstrate superiority of the MIB in comparison with existing Australian OTC labels is currently not readily available. Importantly, the identified improvements suggested by participants may have the ability to increase consumer perceived MIB quality and usability, which may result in increased use of the labels prior to, and during, medicine administration. Nonetheless, improvement of both perceived quality and usability are not replacements for demonstrated label format usability. Therefore, further employment of ‘user testing’, as advocated for use in label development,^19^ is necessary prior to widespread implementation across all OTC medicines in Australia. This iterative approach to label development can both identify and address label shortcomings to yield better performing OTC labels,^45^, ^46^ whilst also providing evidence that the MIB is fit for purpose. Implementation of a standardized OTC label format across all OTC medicines that poorly supports consumers’ ability to find and understand important medicine information, or performs worse than existing OTC labels, could cause harm to those who engage in self‐management.

There are some limitations to this work. As the core study focus was to explore consumer opinions on the MIB, it was not provided as part of complete OTC product packaging, but as single, whole panels affixed to blank boxes. The boxes utilized could be regarded as larger than actual OTC product packaging available with many current OTC products. Accordingly, the perspectives raised may not encompass possible opinions of MIBs presented on different sized packaging or split across multiple panels. This suggests the need for further consultation with consumers to gain insight into consumer opinions of the MIB when it has been included as part of various packaging styles and sizes, representative of the diversity of available OTC products. As this was a qualitative study, these findings also cannot be generalized to the Australian and UK population. However, the results could inform OTC labelling policy and guidelines and also help inform future quantitative evaluations of consumer perceptions of the MIB, in an effort to obtain generalizable data.

## Conclusion

Participants were generally receptive to standardization of OTC labels. Aspects of the MIB that were favoured echoed good information design principles, such as the effective use of bullet points and clear headings. However, a number of improvements were deemed necessary by participants in order to optimize perceived MIB quality and usability; such as, the reordering of information, use of colour, bolding of key terms in relation to warnings information and addition of content including further contact details and inactive ingredient information. Consequently, due consideration should be given to the improvements proposed when optimizing and finalizing the MIB for the purpose of standardization. Consumer opinions are of value and should be taken into account as part of overall OTC label development to help facilitate widespread improvement of OTC label quality, within the overarching aim of promoting safe medication use by consumers.

## Conflict of interest

David K Raynor is the co‐founder and academic advisor for Luto Research, a company that provides performance‐based testing services for health information.

## Funding

Vivien Tong was the recipient of the Royston George Booker Scholarship in 2013, a University of Sydney Grant‐in‐Aid that supported travel necessary for the completion of data collection for the UK arm of this broader project.

## References

[1] Blom AT , Rens JA . Information about over‐the‐counter medication: the role of the pharmacy. Patient Education and Counseling, 1989; 14: 181–189. 10296744 10.1016/0738-3991(89)90032-3

[2] Newby DA , Hill SR , Barker BJ , Drew AK , Henry DA . Drug information for consumers: should it be disease or medication specific? Results of a community survey. Australian and New Zealand Journal of Public Health, 2001; 25: 564–570. 11824997 10.1111/j.1467-842x.2001.tb00327.x

[3] Simoens S , Lobeau M , Verbeke K , van Aerschot A . Patient experiences of over‐the‐counter medicine purchases in Flemish community pharmacies. Pharmacy World & Science, 2009; 31: 450–457. 19333777 10.1007/s11096-009-9293-0

[4] Birchley N , Conroy S . Parental management of over‐the‐counter medicines. Paediatric Nursing, 2002; 14: 24–28. 10.7748/paed.14.9.24.s21 12510331

[5] Gray NJ , Cantrill JA , Noyce PR . ‘Health repertories’: an understanding of lay management of minor ailments. Patient Education and Counseling, 2002; 47: 237–244. 12088602 10.1016/s0738-3991(01)00226-9

[6] Harris interactive for NCPIE (National Council on Patient Information and Education) . Attitudes and beliefs about the use of over‐the‐counter medicines: a dose of reality; a national survey of consumers and health professionals, 2002.

[7] Kelly FS , Williams KA , Benrimoj SI . Does advice from pharmacy staff vary according to the nonprescription medicine requested? The Annals of Pharmacotherapy, 2009; 43: 1877–1886. 19843841 10.1345/aph.1L121

[8] Emmerton L . The ‘third class’ of medications: sales and purchasing behavior are associated with pharmacist only and pharmacy medicine classifications in Australia. Journal of the American Pharmacists Association: JAPhA, 2009; 49: 31–37. 19196594 10.1331/JAPhA.2009.07117

[9] Sleath B , Rubin RH , Campbell W , Gwyther L , Clark T . Physician‐patient communication about over‐the‐counter medications. Social Science & Medicine, 2001; 53: 357–369. 11439819 10.1016/s0277-9536(00)00341-5

[10] Evans SW , John DN , Bloor MJ , Luscombe DK . Use of non‐prescription advice offered to the public by community pharmacists. International Journal of Pharmacy Practice, 1997; 5: 16–25.

[11] Directive 2001/83/EC of the European Parliament and of the Council of 6 November 2001 on the Community code relating to medicinal products for human use, Europe [statute on the Internet]. 2012 Nov 16. Available at: http://ec.europa.eu/health/files/eudralex/vol-1/dir_2001_83_consol_2012/dir_2001_83_cons_2012_en.pdf, accessed 3 May 2015.

[12] Poisons Standard 2014 Schedule 1‐ Standard for the uniform scheduling of medicines and poisons No. 5 October 2014, Australia [Internet]. 2014 Oct. Available at: http://www.comlaw.gov.au/Details/F2014L01343, accessed 3 May 2015.

[13] Aslani P . Consumer medicine information conundrums. Australian Prescriber, 2007; 30: 122–124.

[14] Taylor J , Lo YN , Dobson R , Suveges L . Consumer over‐the‐counter usage and attitudes: a survey in one Canadian city. International Journal of Pharmacy Practice, 2008; 16: 295–302.

[15] Nabors LA , Lehmkuhl HD , Parkins IS , Drury AM . Reading about over‐the‐counter medications. Issues in Comprehensive Pediatric Nursing, 2004; 27: 297–305. 15764435 10.1080/01460860490884192

[16] Sansgiry SS , Cady PS , Patil S . Readability of over‐the‐counter medication labels. Journal of the American Pharmaceutical Association: APhA, 1997; NS37: 522–528. 9479403 10.1016/s1086-5802(16)30244-3

[17] Sansgiry SS , Shringarpure G . Manufacturers’ compliance with the US Food and Drug Administration's Over‐the‐counter Human Drugs: Labeling Requirements. Packaging Technology and Science, 2003; 16: 91–98.

[18] Tong V , Raynor DK , Aslani P . Design and comprehensibility of over‐the‐counter product labels and leaflets: a narrative review. International Journal of Clinical Pharmacy, 2014; 36: 865–872. 24980281 10.1007/s11096-014-9975-0

[19] Communication Research Institute of Australia . Labelling Code of Practice: Designing Usable Non‐Prescription Medicine Labels for Consumers. Canberra: Communication Research Press, 2004: 19 p.

[20] Over‐the‐counter human drugs; labeling requirements. Federal Register [regulation on the Internet], 1999;64:13254–13303. Available at: http://www.gpo.gov/fdsys/pkg/FR-1999-03-17/pdf/99-6296.pdf, accessed 3 May 2015. 10557606

[21] Mendat CC , Watson AM , Mayhorn CB , Wogalter MS . Age differences in search time for two over‐the‐counter (OTC) drug label formats. Proceedings of the Human Factors and Ergonomics Society Annual Meeting, 2005; 49: 200–203.

[22] Shaver EF , Wogalter MS . A comparison of older vs. newer over‐the‐counter (OTC) nonprescription drug labels on search time accuracy. Proceedings of the Human Factors and Ergonomics Society Annual Meeting, 2003; 47: 826–830.

[23] Australian Government Department of Health and Ageing Therapeutic Goods Administration . Australian regulatory guideline for over‐the‐counter medicines Appendix 3: Guidelines on presentation aspects of OTC applications Version 1.0 October 2012. Australian Capital Territory: Therapeutic Goods Administration, 2012: 26 p.

[24] Therapeutic Goods Order No. 69 – General requirements for labels for medicines, Australia [statute on the Internet]. 2014 Jul 14. Available at: http://www.comlaw.gov.au/Details/F2014C00926, accessed 3 May 2015.

[25] Australian Government Department of Health and Ageing Therapeutic Goods Administration . TGA medicine labelling and packaging review: consultation paper version 1.0, May 2012. Australian Capital Territory: Therapeutic Goods Administration, 2012: 55 p. Report No.:R12/759506.

[26] Medicines Act 1968 Chapter 67, UK [statute on the Internet], 1968 Available at: http://www.legislation.gov.uk/ukpga/1968/67/pdfs/ukpga_19680067_en.pdf, accessed 3 May 2015.

[27] Sansom LN (ed.). Australian Pharmaceutical Formulary and Handbook: The Everyday Guide to Pharmacy Practice, 22nd edn Canberra: Pharmaceutical Society of Australia, 2012.

[28] Australian Medicines Handbook. Adelaide: Australian Medicines Handbook Pty Ltd, 2012.

[29] Raynor DK , Dickinson D . Key principles to guide development of consumer medicine information – content analysis of information design texts. The Annals of Pharmacotherapy, 2009; 43: 700–706. 19318595 10.1345/aph.1L522

[30] Britten N . Qualitative interviews In: Pope C, Mays N (eds) Qualitative Research in Health Care, 3rd edn Oxford: Blackwell Publishing Ltd, 2006: pp 12–20.

[31] Sless D , Shrensky R . Writing About Medicines for People: Usability Guidelines for Consumer Medicine Information, 3rd edn North Sydney: Australian Self Medication Industry, 2006.

[32] Green J , Thorogood N . Qualitative Methods for Health Research, 3rd edn London: SAGE Publications Ltd, 2014.

[33] Miles MB , Huberman AM . Qualitative Data Analysis: An Expanded Sourcebook, 2nd edn Thousand Oaks: SAGE Publications, Inc., 1994.

[34] Guest G , Bunce A , Johnson L . How many interviews are enough?: an experiment with data saturation and variability. Field Methods, 2006; 18: 59–82.

[35] Food Standards Australia New Zealand . Nutrition information panels [Internet]. 2012 Jul. Available at: http://www.foodstandards.gov.au/consumer/labelling/panels/Pages/default.aspx, accessed 11 November 2014.

[36] Lokker N , Sanders L , Perrin EM *et al* Parental misinterpretations of over‐the‐counter pediatric cough and cold medication labels. Pediatrics, 2009; 123: 1464–1471. 19482755 10.1542/peds.2008-0854 PMC2911576

[37] Wogalter MS , Vigilante WJ Jr . Effects of label format on knowledge acquisition and perceived readability by younger and older adults. Ergonomics, 2003; 46: 327–344. 12637173 10.1080/0014013021000048006

[38] Vigilante WJ Jr , Wogalter MS . Over‐the‐counter (OTC) drug labeling: format preferences. Proceedings of the Human Factors and Ergonomics Society Annual Meeting, 1999; 43: 103–107.

[39] Vigilante WJ Jr , Wogalter MS Jr . The preferred order of over‐the‐counter (OTC) pharmaceutical label components. Therapeutic Innovation & Regulatory Science, 1997; 31: 973–988.

[40] Aikin KJ . Consumer comprehension and preference for variations in the proposed over‐the‐counter drug labeling format: final report. USA: Center for Drug Evaluation and Research, Food and Drug Administration, 1998.

[41] Andre AD , Wickens CD . When users want what's not best for them. Ergonomics in Design: The Quarterly of Human Factors Applications, 1995; 3: 10–14.

[42] King JP , Davis TC , Bailey SC *et al* Developing consumer‐centered, nonprescription drug labeling a study in acetaminophen. American Journal of Preventive Medicine, 2011; 40: 593–598. 21565649 10.1016/j.amepre.2011.02.016

[43] Sless D . Comments‐ TGA medicine labelling and packaging review [Internet]. Communication Research Institute, 2012 Available at: http://tga.gov.au/sites/default/files/consult-labelling-packaging-review-120524-submission-cri.pdf, accessed 22 December 2014.

[44] NPS MedicineWise . Find the active ingredient [Internet]. 2012 Oct 25. Available at: http://www.nps.org.au/topics/how-to-be-medicinewise/managing-your-medicines/find-the-active-ingredient, accessed 21 November 2014.

[45] Rogers D , Shulman A , Sless D , Beach R . Designing better medicine labels: report to PHARM. Canberra: Communication Research Institute of Australia, 1995: 69 p.

[46] Sless D , Tyers A . Medicine Labelling for Consumers. Australia: Communication Research Institute of Australia.

